# Reducing levels of medical device contamination through package redesign and opening technique

**DOI:** 10.1371/journal.pone.0206892

**Published:** 2018-11-07

**Authors:** Paula Perez, Tamara Reid Bush, Hyokyoung G. Hong, Wu Pan, Larissa Miller, Laura Bix

**Affiliations:** 1 School of Packaging, Michigan State University, East Lansing, MI, United States of America; 2 Mechanical Engineering Dept., Michigan State University, East Lansing, MI, United States of America; 3 Department of Statistics and Probability, Michigan State University, East Lansing, MI, United States of America; 4 Advanced Chronic Nursing Care, Nursing Program, Lansing Community College, Lansing, MI, United States of America; Thomas Jefferson University, UNITED STATES

## Abstract

**Objectives:**

The goal of this research was to evaluate how material curl, package structure and handling of pouches containing medical devices affect rates of contact between non-sterile surfaces and sterile devices during aseptic transfer.

**Methods:**

One hundred and thirty-six individuals with practical experience in aseptic technique were recruited. Participants were asked to present the contents of four different pouch designs (a standard, one designed to curl in, another to curl out and one that incorporated a tab) using two transfer techniques. During the first block of trials “standard technique” was used; participants presented using their typical methods to the sterile field. Trials in the second block employed “modified technique”; participants were instructed to grab the package at the top center and present package contents using a single, fluid motion. The outside of the pouch and the backs of the participants’ hands were coated using a simulated contaminant before each trial. The simulant was undetectable in the visible spectrum, but fluoresced under a black light. The dependent variable was recorded in a binary fashion and analyzed using a generalized linear mixed model.

**Results:**

Participants were between 20–57 and the averaged year 5.1 years of experience in aseptic technique. The data analysis was based on generalized linear mixed effects (GLMM) model, which accommodates the repeated measurements within the same participant. The effect of the pouch design was significant (P‹0.001), but the effect of aseptic technique did not suggest significance (P = 0.088). Specifically, pouches designed with the material curled outward resulted in significantly fewer contacts with non-sterile surfaces than the other styles, including the inward, tab, and standard styles; this was true regardless of the used aseptic technique, standard (P = 0.0171, P = 0.0466, P = 0.0061, respectively) or modified (P‹0.0001 for all comparisons)).

**Conclusion:**

Results presented here contribute to a growing body of knowledge that investigates packaging as a potential route of contamination for sterile devices during aseptic presentation. Specifically, we provide insights regarding how both package design and opening technique can be informed in ways that build safety into the healthcare system.

## Introduction

Healthcare associated infections (HAIs), infections patients get while receiving medical treatment, have been categorized among the ten leading causes of morbidity and mortality in the United States [1]. In 2011, 722,000 cases of HAIs were reported [2], and it has been reported that one in every 25 patients in acute care hospitals have acquired an HAI [2]. These infections not only result in illness and suffering, they also impose an economic burden due to prolonged lengths of stay (LOS), loss of productivity and added costs associated with patients’ medical treatments.

HAIs can be transmitted directly, from a single person to the patient, or indirectly, through an intermediate object or person [3]. One potential indirect pathway for an infectious agent to be transferred to a patient is when a sterile medical device touches a non-sterile surface during the process of being transferred to the sterile field.

Despite the fact that sterile medical devices experience a sterilization process, only the inside portion of the package is sterile. This is because during transportation, handling and storage, packages are exposed to non-sterile environments. As a result, if the sterile device contacts the outside of the package, or the hands of the person transferring it, the potential for an indirect transfer of microbes exists. Of further concern is the finding that some microorganisms, such as methicillin resistant *Staphylococcus aureus* (MRSA), can survive on packaged goods for more than 38 months [4].

When medical devices serve as a vehicle for indirect transmission, the initial contamination tends to occur from a small number of micro-organisms that are transferred to the device, either from healthcare provider’s hands or skin [5] or other environmental sources [6]. Device-associated HAIs present themselves in a variety of ways. Richards et al. (2000) suggests that bloodstream, urinary track and respiratory infections occurring in medical-surgical intensive care units were positively associated with the use of invasive devices [7]. In another analysis encompassing 1,022 HAIs, 12% were associated with a medical device [8].

Surgical Site Infections (SSI), are common complications in acute care and are the third most commonly reported type of HAI, accounting for 14%-16% of HAIs among hospital patients and occurring in 2–5% of patients undergoing inpatient surgery [9].

Recognizing the paramount role of the sterile field in preventing infections of all types, the Association of peri-Operative Registered Nurses (AORN) [10]^,^[11] and the Association of Surgical Technologists (AST) [12] have both developed standards regarding its creation and maintenance. These standards, in part, addresses package opening and transfer of devices to the field. PeriOperative personnel are trained in procedures (i.e. package opening and transfer of devices) using methods intended to avoid device contamination [13] in an approach termed “aseptic technique.” The ultimate goal is to reduce the incidence of device contamination during transfer. That said, the organizations differ on some aspects of their guidelines for aseptic technique, and opening techniques are not widely standardized across all hospitals[14].

AST standards suggest that small peel packs should be opened and flipped on to the sterile field using aseptic technique with the “glue area” of the package considered as the boundary between sterile and non-sterile areas [12]. In contrast to AST recommendations, AORN indicates that two people should use a technique commonly referred to as a “pick.” In this technique a circulator, someone preparing the room that is not considered sterile, handling only the outside of the package, would present the contents to someone who was “scrubbed” (considered sterile) so that they could carefully remove the contents without touching anything that was not sterile. AORN’s reasoning for this approach is, “peel pouches should be presented to the scrubbed person to prevent contamination of the contents (i.e. the medical device) as the edges of the package *may curl* [emphasis added] and the contents of the package may touch unsterile edges” [10].

Although it is reasonable to assume that curling edges of the pouch could contaminate sterile contents, evidence is needed to validate that assumption, and a search of the literature yields little work regarding the relationship between packaging design, transfer technique and contamination. The available, published literature focuses on operating room traffic [15] and package integrity [16]. Within our scope (packaging as a potential vehicle of microbe transfer), a dearth of information is available.

Crick et al. (2008) suggested that removal of a sterile inner package from a double barrier resulted in increased rates of contamination. They suggest that contact with the outer/non-sterile portion of the package and increased rates of handling induced by double barrier systems led to higher contamination rates as compared with products packaged in a single barrier system [17]. Similarly, Smith et al. (2009) studied the probability of contamination of the sterile field when airborne contaminants settled after high opening forces and handling scattered them [18]. Trier et al. (2014) studied how package size impacted contamination rates during aseptic technique. Researchers concluded that large packages induced higher rates of contact with non-sterile surfaces compared to transfers that utilized smaller packages (P = 0.0130). Authors also suggested that the number of hand repositionings increased with increasing pouch size [19]. Hopper et al. (2010) indicated that corners of flexible pouches “might curl into package,” contaminating the product, which would be considered a break in sterile technique [20].

In summary, the literature suggests that packaging and aseptic technique both have the potential to play a role in contamination; yet little work has been done to objectively characterize factors that facilitate (or hinder) successful transfers to the sterile field.

## Objective

### Overarching goal

The goal of this research was to evaluate how material curl, package structure and handling of pouches for medical devices affect rates of contact between non-sterile surfaces and sterile devices during aseptic transfer.

### Proximal goals

- Evaluate how modified technique to device transfer during asepsis impacts rates of contact between sterile device and non-sterile surfaces
- Evaluate how two packaging design factors, namely, material curl and package structure influence rates of contact between non-sterile surfaces and sterile devices during sterile transfer.

## Materials and methods

### Volunteer subjects

To participate in the study, individuals were required to: be at least 18 years old; have no known history of a skin condition (e.g. eczema, latex allergy, etc.); have a history of employment as a healthcare professional with experience in aseptic technique, or be a healthcare student with practical experience in aseptic technique; and be willing to be videotaped presenting devices to a simulated sterile field. All methods were conducted in accordance with procedures approved under the Social Science/Behavioral Educational Institutional Review Board (SIRB #15–1199).

Testing was conducted at four different locations through affiliated health systems programs, namely: Lansing Community College (Lansing, MI), Baker College (Clinton Twp., MI), Grand Valley State University (Grand Rapids, MI), Sparrow Hospital (Lansing, MI), and Michigan State University (East Lansing, MI). The research team worked with healthcare professionals and teaching faculty to distribute flyers and e-mails.

Written consent was obtained from all participants. Following the consent process, participants were asked to provide basic demographic information, such as age, gender, and information regarding their professional experience as a healthcare provider.

### Simulating contamination

**Fig 1:**
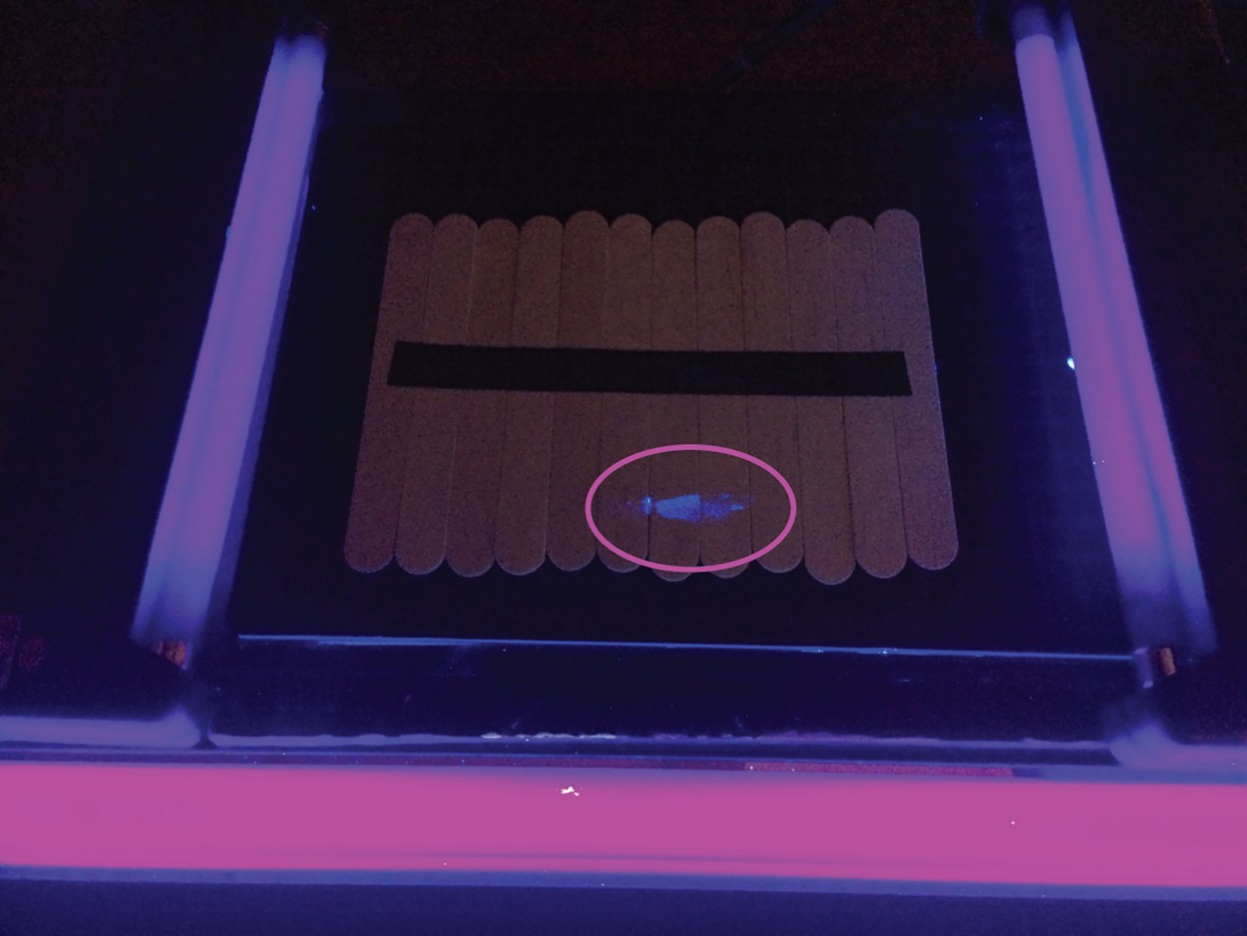
UV light configuration showing contamination on the device (contamination is highlighted with circle).

To detect contamination, methods first proposed by *Crick et al*. [17] and adapted by *Trier et al*. [19] were employed to identify contact between package contents with non-sterile surfaces, specifically the provider’s hands and the outside of the pouch. Glitterbug, a lotion commonly used in infection control programs as a model for germ transfer in classes on hand hygiene (Brevis Corporation, Salt Lake City, UT), was applied to the outer portion of the pouch and the gloves of the healthcare provider. The lotion is not visible unless exposed to an ultra violet light source. After the device was transferred to the sterile field, it was scanned with a UV light (Brevis Corporation, Salt Lake City, UT) inside a black tent H1900 (ePhotoInc Hayward, CA) to detect transfer of Glitterbug. Presence of the Glitterbug analyte was indicative of contact with the outside of the pouch or hands (i.e., nonsterile surfaces). Configuration of the lights and samples within the tent are presented in Fig 1; contamination was recorded for each trial as researchers viewed them and each sample was photographed (once on each side) to enable post-hoc review of sample contamination.

### Device

Tongue depressors were used to model the device transferred to the field. They were chosen because they represent a low-cost, easily-accessible medical device with a porous structure that allows for the ready transfer of the simulated contaminant. Tongue depressors were taped together horizontally on both sides of the mock device using black electric tape (see Fig 1); this prevented them from flowing or tumbling as individual components during their transfer.

### Pouch treatments

#### Standard pouch

**Fig 2:**
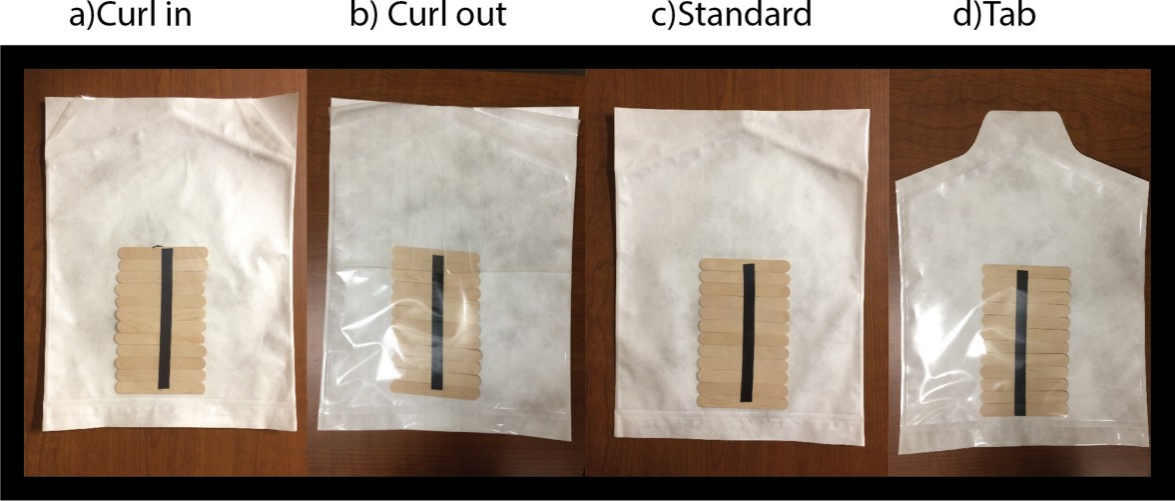
Pouch designs.

A standard chevron pouch (see Fig 2) sized, 40.64 cm x 45.72 cm manufactured from Allegro T, 48 ga PET/28.8# material by Rollprint, Packaging Products (Addison, IL) comprised one of the four treatments used in the study, namely the standard treatment. This design was used as the design base to create the three other treatments tested: (1) a variation with an inward curl, (2) a variation with an outward curl, and (3) an altered physical structure comprised of a single tab in the top center (no curl induced).

#### Creation of inward curl

To induce inward curling (See Fig 2A), we utilized a Sencorp dual shuttle tray sealer Model MD2420, Serial No.: 015 outfitted with a customized flat plate (400 Kidds Hill Rd. Hyannis, MA 02601). The pressure component was eliminated, allowing the team to use the equipment as a consistent heat source. The heat sealer was set to a temperature of 250 ^o^C with a five second dwell time. The pouch was placed on the flat plate horizontally (opening area facing the left side and film portion closest to the heated platen). After the five-second exposure time, the pouch was removed and stored vertically.

#### Creation of outward curl

**Fig 3:**
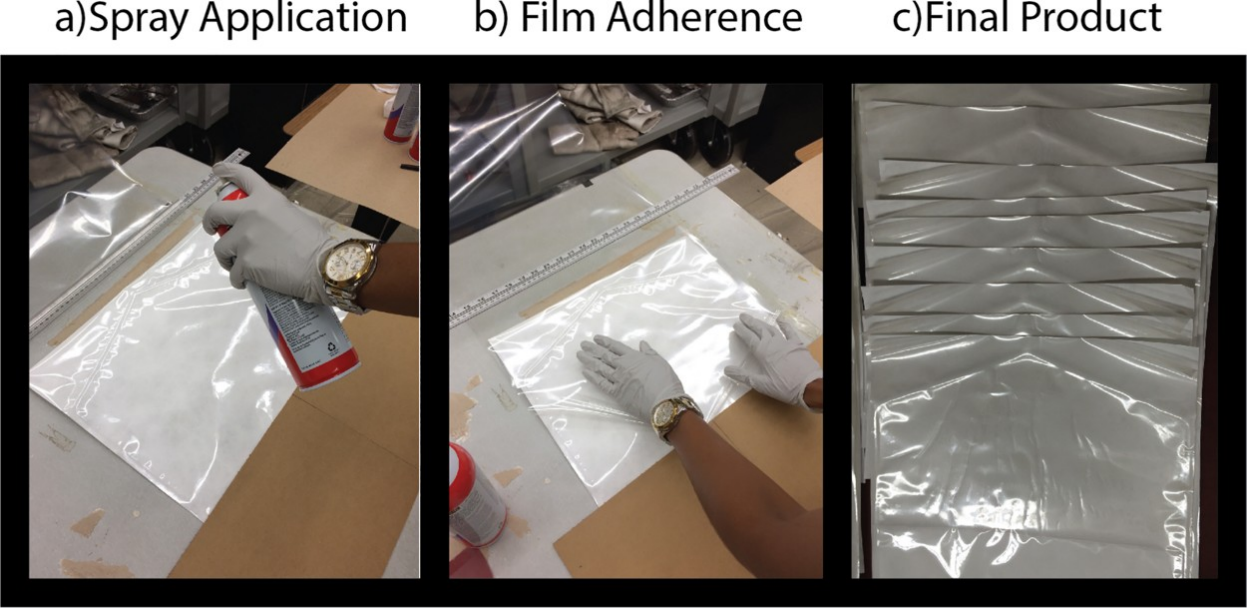
Assembly of outward curling pouch.

To induce the outward curling pouch (See 2B), an extra layer of film CLEARFOIL M3 manufactured by Rollprint, Packaging Products (Addison, IL) was adhered to the top half, namely the half that included the chevron feature, of the outer layer of the film side of the standard pouch (see Fig 3). The extra layer was adhered using a Super 77 multipurpose adhesive spray manufactured by Scotch, USA so that the added film curled away from the opening area (see Fig 3A). The material was cut in the same shape and size as the original package (see Fig 3B and 3C).

#### Creation of pouches with a pull tab feature

**Fig 4:**
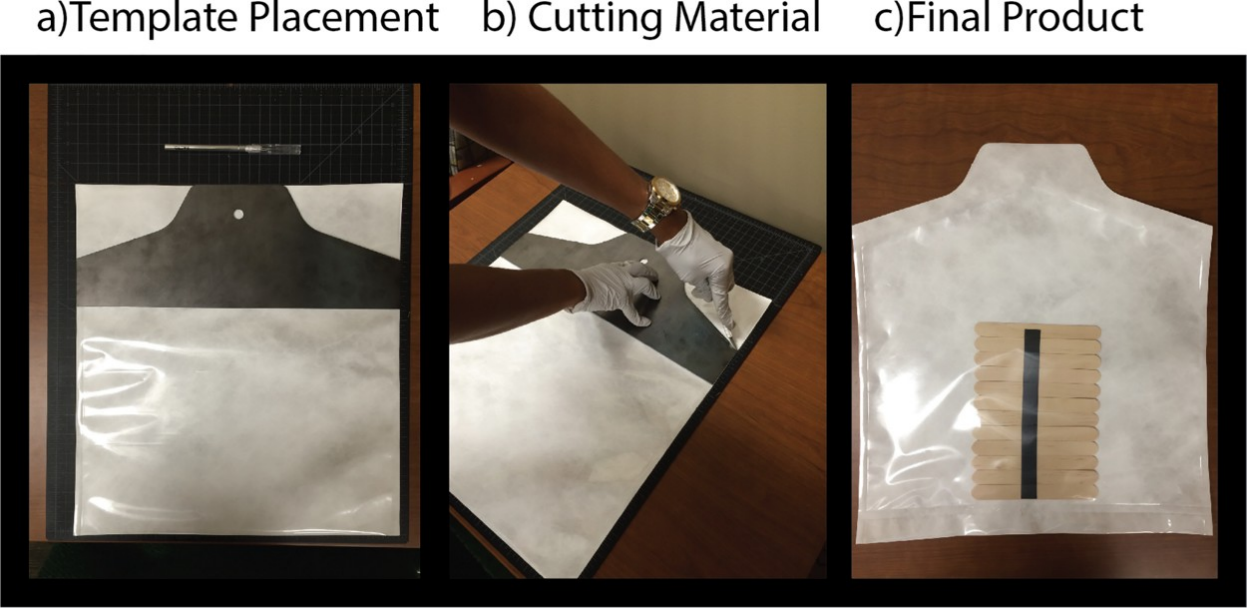
Production of single tab pouch.

To create the treatment comprised of a single pull tab (See Fig 2D), the pouch manufacturer (Rollprint Packaging Products; Addison, IL) provided a gripping area that was larger than what is typically employed; an additional 3.81cm of extra material in the chevron area of each pouch. A metal cutting template was machined from steel so that the extra material was cut in the form of a tab (see Fig 4A) To produce consistent pouches, researchers laid the template over each pouch and cut around the template with an Exacto-style blade (see Fig 4A and 4B).

All pouches were filled with a set of tongue depressors and sealed using a Sencorp Pouch Sealer Model 24AS/1 Serial No. 06–04236, (400 Kidds Hill Rd. Hyannis MA 02601). The parameters used were: 250 ^o^F, three seconds, and 50 pounds per square inch (PSI).

### Data collection

For each opening trial, participants were provided a new set of gloves (Fingertip Textured Flexal Nitrile Powder-Free Exam Gloves manufactured for Cardinal Health, Waukegan, IL). Height adjustable tables (38.1 cm x 76.2 cm) were used to simulate the sterile field. Participants were asked to adjust the table to their desired height prior starting the experiment. The exterior portion of the package and the dorsum side of the hand for each participant were coated with Glitterbug (Brevis Corporation, Salt Lake City, UT) to serve as a simulant for contamination. Application of the lotion excluded the provider’s finger pads to reduce the likelihood of changing the frictional relationship between the providers’ hands and the pouch.

Order of treatments (inward, outward, tab and standard; each appearing twice within a block) was randomized within two blocks, for a total of 16 openings per participant. For the first block of eight openings, participants were instructed to, “transfer the contents onto the sterile field using aseptic technique;” this was termed “standard aseptic technique.” For the second block of openings, participants were instructed to “grasp each pouch at the center top, pull the package apart in one large movement, and transfer the contents onto the sterile field using aseptic technique.” This approach was termed “modified aseptic technique;” blocking was done on technique in order to prevent participants from being influenced by the research team’s opening instructions. To ensure that standard vs modified aseptic techniques were indeed distinct for each test subject and that crossovers did not occur in our experiment, we closely monitored when undertook standard and modified aseptic techniques.

To avoid cross-contamination during the preparation and testing of the samples, two teams of researchers were used; “dirty” personnel were responsible for coating pouches as well as applying the simulated contaminant to the gloved hands of the participants. In contrast, the “clean” team was in charge of: recording trial results; reading opening instructions; replacing drapes between trials; transferring, scanning and documenting samples using cameras and data sheets in the black tent. Each tent was equipped with a Canon Power Shot camera set to high speed burst with flash deactivated; the rest of the settings were kept as default.

An exit survey was administered upon completion comprised of three questions. (1) Of the packages you opened today, were any more challenging to open than others? If so, could you point them out and indicate what about them made opening more challenging? (2) Of the packages you opened today, were any easier to open than others? If so, could you point them out and indicate what about them made them easier to open? (3) Do you have any other comments you would like to share about the packages, or about the study?

### Statistical methods

Data were recorded as a binary response (contamination: yes/no) and analyzed using generalized linear mixed model fitted with a logit link function assuming a Bernoulli distribution. Demographic information (e.g. sex, age, handedness, etc.) and years of aseptic experience were included on the initial model for evaluation. However, the final model excluded demographic factors since they did not suggest significance. Factors included in the linear predictor model were: treatment (inward, outward, standard, tab), and aseptic technique (standard, modified). We also included interaction between treatment and aseptic technique. To avoid confusion, we denote the standard pouch design as STD when presenting the results in the following section. Treatment and aseptic technique were included as fixed effects. To accommodate repeated measurements within the same subject effects were modeled as a random effect. Data analysis was performed using PROC GLIMMIX of SAS (Version 9.4 TS Level 1M1 SAS Institute Inc. NC) implemented using Newton-Raphson with ridging as the optimization technique. Pairwise comparisons were conducted across different subgroups using Bonferroni adjustments, as it is considered conservative method that protects against Type I error inflation since multiple comparisons between different treatments were made. Results were presented as least squares means (LSMEAN) and standard error mean (SEM). The α level was set at 0.05.

## Results

### Participant demographics

A total of 137 data sets were collected. One of the participants participated twice; as a result, one data set was removed. Another participant wrote 0 as years of experiment in aseptic technique; however, experience in aseptic technique was a condition of the study, therefore, data collected from this subject was also removed. Seven more data sets were removed as incomplete. As such, the final analysis comprised a total of 128 completed datasets contributed by 19 male participants and 109 female participants. Females ranged in age from 20–57 (Mean = 29.72; Standard Deviation (SD) = 7.78) and males from 25 to 51 years (Mean = 38.37; SD = 8.78). Participant’s occupations were: Certified Nursing Assistants (CAN or CENA; 6.25%), Certified Surgical Technologists (CST; 14.84%) Surgical Technologists (ST; 3.13%) Emergency Medical Technicians (EMT; 1.56%), Physical Therapists (3.13%), nurse (Licensed Practical: 17.97%; Registered Nurse: 11.72; others: 1.56%), Students (Physical Therapists students: 2.34%; RN students: 2.34%; Nursing students: 21.09%; others: 9.38%), Paramedics (2.34%), Phlebotomists (0.78%), and others (1.57%). Participants averaged 5.07 (SD = 7.44) years of experience in aseptic technique, which ranged from 5 months to 36 years.

### Effect of pouch design

**Fig 5:**
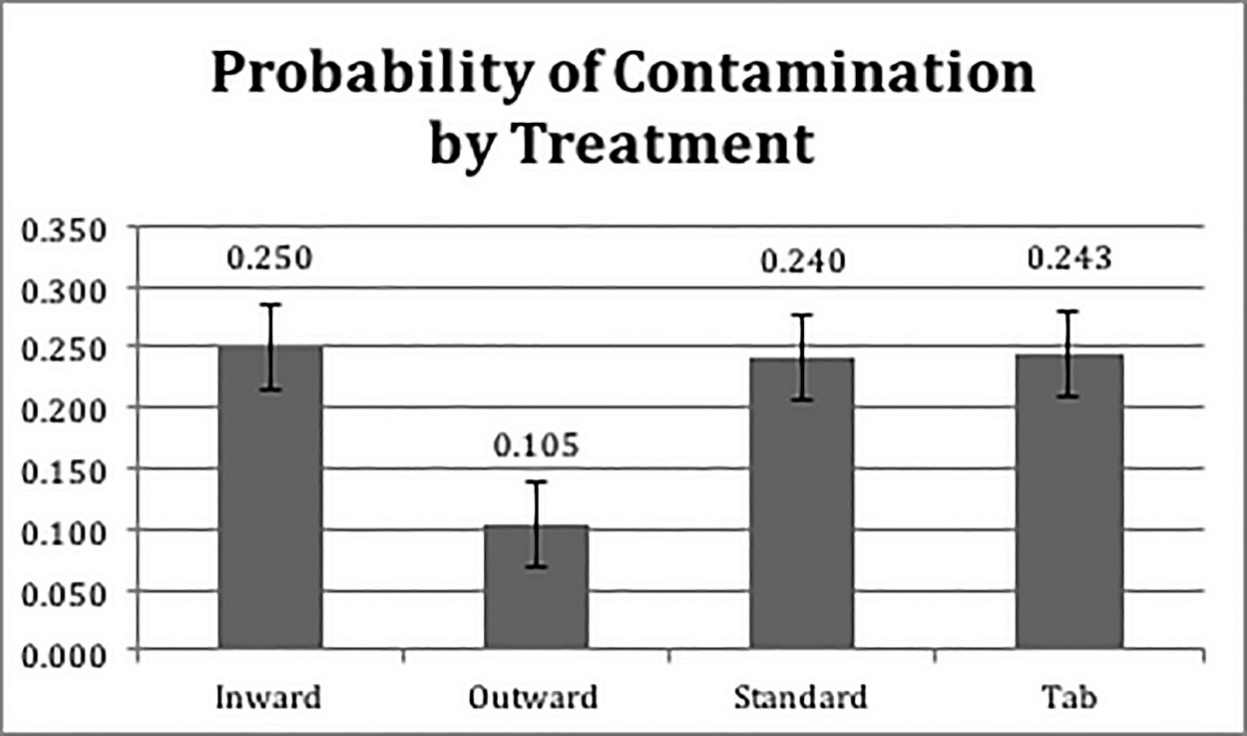
Probability of contamination by treatment.

**Fig 6:**
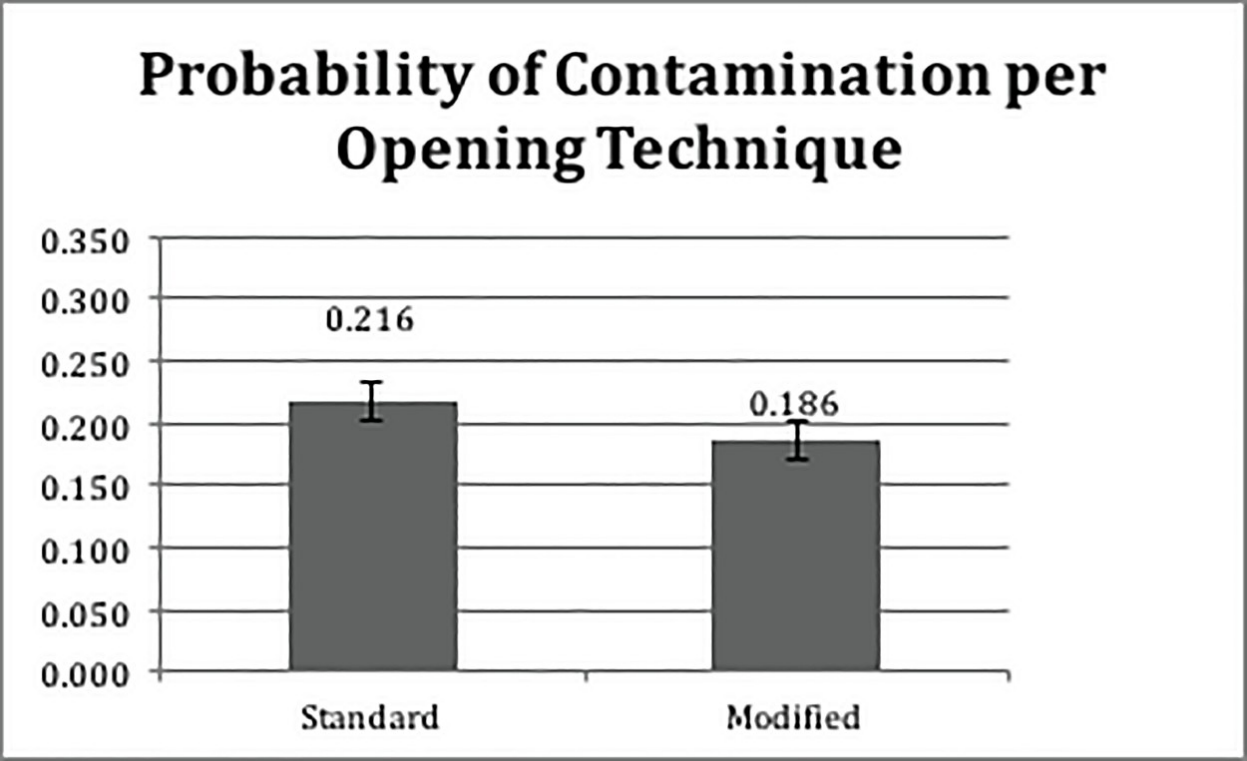
Probability of contamination per opening technique (standard or modified “one pull”).

The GLMM results indicated a significant effect of the pouch design (P <0.001) but did not yield evidence of a statistically significant effect of aseptic technique (P = 0.0880). Figs 5 and 6 show the estimated probability of contamination for each pouch design and opening technique, respectively. The two-way interaction between pouch design and aseptic technique was non-significant (P = 0.0983). Table 1 reports the detailed results of the GLMM.

**Table 1:** Generalized linear mixed model (GLMM) results for the effects of aseptic technique and pouch designs on contamination.

**Solutions for fixed effects**				
**Effect**	**Estimate**	**Standard error**	**t-value**	**P-value**
Intercept	-0.8776	0.1569	-5.60	<0.0001
**Aseptic technique**				
Standard	-0.2049	0.2038	-1.01	0.3150
Modified	∙	∙		∙
**Pouch designs**				
Inward	-0.0631	0.2006	-0.31	0.7533
Tab	-0.1294	0.2026	-0.64	0.5231
Outward	-0.7191	0.2186	-3.29	0.0010
STD	∙	∙		∙
**Aseptic technique ×Pouch designs**				
Inward**×**Standard	0.2439	0.2862	0.85	0.3942
Inward**×**Modified	∙	∙		∙
Tab**×**Standard	0.2984	0.2879	1.04	0.3001
Tab**×**Modified	∙	∙		∙
Outward**×**Standard	-0.4761	0.3661	-1.42	0.1567
Outward**×**Modified	∙	∙		∙
STD**×**Standard	∙	∙		∙
STD**×**Modified	∙	∙		∙
**Tests of fixed effects (Type III)**				
**Effect**	**Degrees of freedom**		**F value**	**P-value**
Aseptic techniques	1		2.91	0.0880
Pouch designs	3		15.15	<0.0001
Aseptic techniques**×**Pouch designs	3		2.10	0.0983

**Fig 7:**
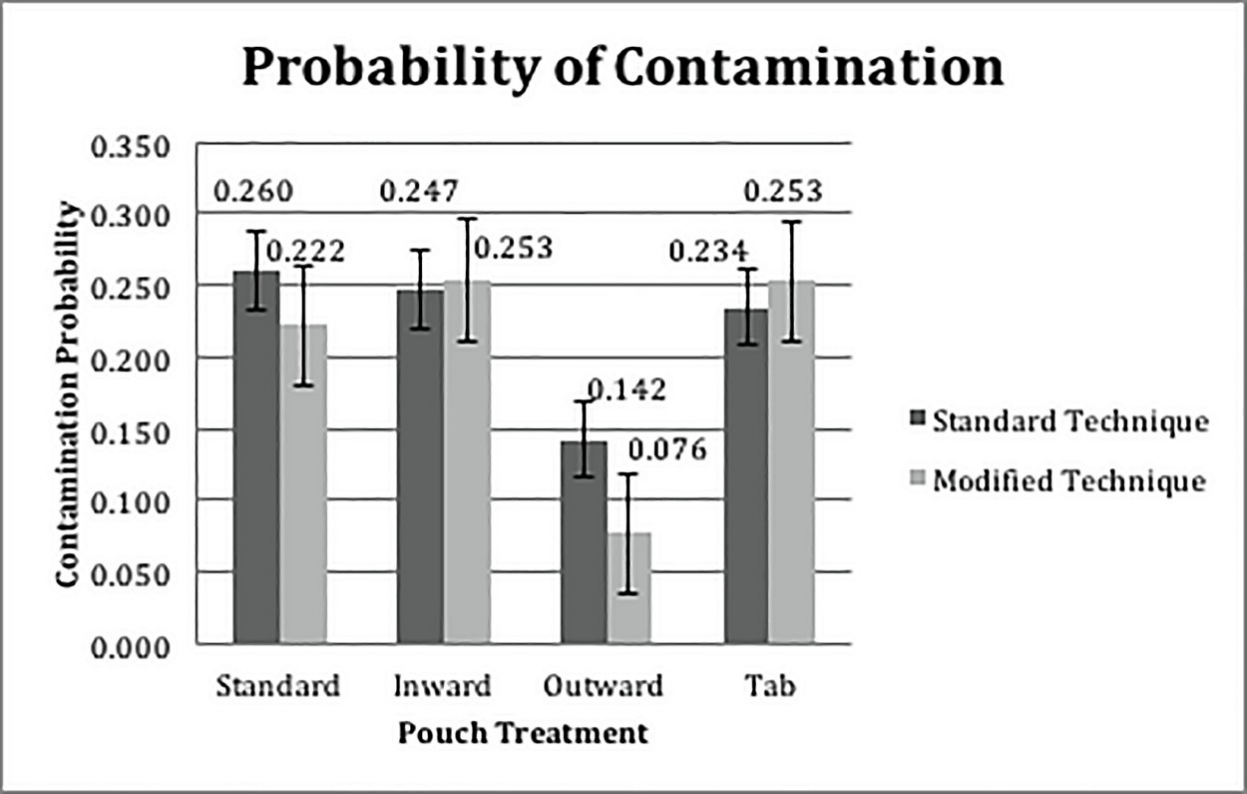
Probability of contamination per opening technique (standard or modified “one pull”).

We also examined rates of contact with non-sterile surfaces by pouch design (see Table 2) and opening technique (see Fig 7).

**Table 2:** Least squares mean estimates with Bonferroni adjustment to examine potential effects on rates of contact with non-sterile surfaces by pouch design.

**Comparison between Outward designs vs. other styles**			
**Effect**	**LSMEAN**	**SEM**	**Adjusted P-value**
Outward vs. Inward	-1.0160	0.1675	<0.0001
Outward vs. Tab	-0.9770	0.1682	<0.0001
Outward vs. STD	-0.9572	0.1683	<0.0001
**Comparison between Outward designs vs. other pouch styles for Modified technique**			
**Effect**	**LSMEAN**	**SEM**	**Adjusted P-value**
Outward vs. Inward	-1.3761	0.2526	<0.0001
Outward vs. Tab	-1.3643	0.2530	<0.0001
Outward vs. STD	-1.1952	0.2556	<0.0001
**Comparison between Outward designs vs. other pouch styles for Standard technique**			
**Effect**	**LSMEAN**	**SEM**	**Adjusted P-value**
Outward vs. Inward	-0.6550	0.2195	0.0171
Outward vs. Tab	-0.5897	0.2213	0.0466
Outward vs. STD	-0.7191	0.2186	0.0061
**The mean difference between aseptic designs for each pouch design**			
**Effect**	**LSMEAN**	**SEM**	**Adjusted P-value**
Inward	0.0391	0.2009	1.000
Tab	0.0936	0.2033	1.000
Outward	-0.6810	0.2673	0.0437
STD	-0.2049	0.2038	1.000

Specifically, when using the standard aseptic technique, outward design induced lower rates of contact between devices and non-sterile surfaces compared to other pouch designs. The least squares estimates of the differences between the mean of the subgroup of the standard aseptic technique with outward design and the subgroups of the standard aseptic technique with inward, tab, and STD were -0.6560±0.2195 (P = 0.0171), -0.5898±0.2213 (P = 0.0466), -0.7191±0.2186 (P = 0.0061), respectively. Similarly, when using modified aseptic technique, the result indicated that the outward design induced lower contamination rates compared to the inward, tab and STD pouch designs. The least squares estimates of the differences between the means of the subgroups were -1.3761±0.2526 (P<0.0001), -1.3643±0.2530 (P<0.0001), -1.1952±0.2556 (P<0.0001), respectively. In terms of the contamination rate, the effect of the aseptic opening technique was significant within the outward treatment group (-0.6810±0.2673, P = 0.0437). Interestingly, although the tab design did not result in a lower contamination rate, 70% of participants indicated it as the preferred design due to ease of opening in the exit survey.

## Conclusions

The literature suggests that large pouches and increased rates of handling during opening are associated with higher contamination rates [19]. We empirically investigated how specific aspects of design and opening approach affected the likelihood of a device contacting the hands of the provider or outside of the package (each of which are not considered sterile) during aseptic transfer. During aseptic transfer, pouches that had outward curling of material resulted in significantly lower contamination rates. Intuitively, this is likely because in these treatments the edges of the package curled away from the opening area, reducing the likelihood of contact between package contents with the unsterile portion of the package, the outside. This design also resulted in even lower contamination rates when participants were instructed to grip the package in the center and use a single, fluid motion to open and dump the contents (modified technique). Standard designs and those where inward curling was induced resulted in significantly high contamination rates.

While the research team hypothesized that the use of a large tab would encourage a center grip with a single pull, would have less excess material and, as such, would result in lower rates of contact between devices and non-sterile surfaces, results suggested the opposite to be true. It is possible that the sides of package curl in, in the tabbed pouches, causing contamination. Despite the fact that the proportion of contaminated trials were similar to those obtained in trials with the standard pouch, 70% of the participants indicated it to be the best design for aseptic presentation when asked in the exit survey. Additionally, 40% of the participants expressed that the standard design was hard to open because it did not provide enough material to grab and open from the center.

## Limitations

The study was executed in contexts that do not represent the stresses of an operating room, which might affect opening behavior. Future study should be conducted to understand how the stresses of different environments affect opening behavior and the likelihood of contamination of sterile devices.

The authors acknowledge that the size of the pouch is large for the presented contents; however, this was chosen as a worst-case representation. In an effort to maintain consistency of the coating process, consistent amounts of Glitterbug were applied to the pouch and provider hands; however, the relationship between this rate of coating and microbial loads presented on the outside of packages has not been characterized. As a result, a positive transfer only represents contact with a non-sterile surface; it doesn’t necessarily indicate an infection.

Additional factors that likely contribute to the likelihood of contact with sterile surfaces require further study. Our intention was to characterize how opening technique and two package design factors (material curl and physical structure) impact contact with non-sterile surfaces during aseptic transfer to the sterile field. To do this, we used a simple structure that was porous in nature to model the medical device. Further investigation is needed to understand how device properties (e.g. center of gravity, weight, flexibility, etc.) affect transfers.

It is also important to note that contact with a non-sterile surface (e.g. the hand of the person presenting the device or the outside of the package) is not the only way a device might become contaminated, and, as such, become a potential vehicle for indirect transmission of a microbe. An additional way that transfer might occur via the packaging is through airborne transfer of the microbes to the field or the device itself. Indeed, Smith’s work [18] suggested a relationship force and scattering of microbes on the sterile field illuminate this as a rich area for future work; that said, it was beyond the scope of our work, which focused on the relationship of packaging and the likelihood of contact with non-sterile surfaces only.

Although, the “Tab” design was preferred by a large number of participants because of ease of opening, it resulted in higher contamination rates. Future study should be conducted to investigate how the addition of a curling outward layer might reduce the contamination rates with a tab design.

## Supporting information

S1 File Raw data- contamination study. (XLSX)

S2 File Raw data- exit survey. (XLSX)
